# A Review on the Expression Pattern of Non-coding RNAs in Patients With Schizophrenia: With a Special Focus on Peripheral Blood as a Source of Expression Analysis

**DOI:** 10.3389/fpsyt.2021.640463

**Published:** 2021-06-18

**Authors:** Soudeh Ghafouri-Fard, Reyhane Eghtedarian, Mohammad Taheri, Annette Beatrix Brühl, Dena Sadeghi-Bahmani, Serge Brand

**Affiliations:** ^1^Department of Medical Genetics, Shahid Beheshti University of Medical Sciences, Tehran, Iran; ^2^Skull Base Research Center, Loghman Hakim Hospital, Shahid Beheshti University of Medical Sciences, Tehran, Iran; ^3^Psychiatric Clinics, Center for Affective, Stress and Sleep Disorders, University of Basel, Basel, Switzerland; ^4^Exercise Neuroscience Research Laboratory, The University of Alabama at Birmingham, Birmingham, AL, United States; ^5^Sleep Disorders Research Center, Kermanshah University of Medical Sciences, Kermanshah, Iran; ^6^Substance Abuse Prevention Research Center, Health Institute, Kermanshah University of Medical Sciences, Kermanshah, Iran; ^7^Division of Sport Science and Psychosocial Health, Department of Sport, Exercise and Health, University of Basel, Basel, Switzerland; ^8^Department of Psychiatry, School of Medicine, Tehran University of Medical Sciences, Tehran, Iran

**Keywords:** lncRNA, miRNA, review, schizophrenia spectrum disorder, development of schizophrenia

## Abstract

Schizophrenia is a destructive neuropsychiatric disease with a median prevalence of 4.0 per 1,000 during the whole life. Genome-wide association studies have shown the role of copy number variants (generally deletions) and certain alleles of common single nucleotide polymorphisms in the pathogenesis of schizophrenia. This disorder predominantly follows the polygenic inheritance model. Schizophrenia has also been linked with various alterations in the transcript and protein content of the brain tissue. Recent studies indicate that alterations in non-coding RNAs (ncRNAs) signature underlie a proportion of this dysregulation. High throughput microarray investigations have demonstrated momentous alterations in the expression of long non-coding RNAs (lncRNA) and microRNAs (miRNAs) in the circulation or post-mortem brain tissues of patients with schizophrenia compared with control samples. While Gomafu, PINT, GAS5, TCONS_l2_00021339, IFNG-AS1, FAS-AS1, PVT1, and TUG1 are among down-regulated lncRNAs in schizophrenia, MEG3, THRIL, HOXA-AS2, Linc-ROR, SPRY4-IT1, UCA1, and MALAT1 have been up-regulated in these patients. Moreover, several miRNAs, such as miR-30e, miR-130b, hsa-miR-130b, miR-193a-3p, hsa-miR-193a-3p, hsa-miR-181b, hsa-miR-34a, hsa-miR-346, and hsa-miR-7 have been shown to be dysregulated in blood or brain samples of patients with schizophrenia. Dysregulation of these transcripts in schizophrenia not only provides insight into the pathogenic processes of this disorder, it also suggests these transcripts could serve as diagnostic markers for schizophrenia. In the present paper, we explore the changes in the expression of miRNAs and lncRNAs in patients with schizophrenia.

## Introduction

Schizophrenia is a destructive neuropsychiatric disease with a median prevalence of 4.0 per 1,000 during the whole life and a lifetime morbid risk of 7.2 per 1,000 (1). The onset of disorder is usually in youth or early adulthood (2) and rarely after the forties or in the childhood (3, 4). In spite of comparable prevalence in both sexes (1), the disorder is usually begins earlier with a more severe course in male subjects (2, 5). The disorder is associated with an increased mortality rate (1) due to suicide and cardiovascular comorbidities. Schizophrenia is diagnosed by the detection of symptoms in three major domains: positive symptoms, such as hallucination, delusion, and disorganized thought and movement, negative symptoms, i.e., lack of interest and enthusiasm, poverty of content of speech, lack of motivation or aptitude to do tasks and affective annihilation, and cognitive symptoms including deficiencies in executive function and attention (6). Genome-wide association studies have shown the role of copy number variants (generally deletions) and certain alleles of common single nucleotide polymorphisms (SNPs) in the pathogenesis of schizophrenia. This disorder predominantly follows the polygenic inheritance model with a significant level of overlap in genetic factors with other psychiatric disorders namely autism and bipolar disorder (7).

Schizophrenia has also been associated with dysregulation of several transcripts and proteins in the brain tissue. These alterations are caused by an intricate dysregulation of gene expression and protein synthesis. Both spatial and temporal elements contribute in determination of this dysregulation. Several studies indicate that alterations in non-coding RNAs (ncRNAs) signature underlie the mechanisms of this dysregulation (8). Changes in expression profile of ncRNAs in the brain tissues of patients with schizophrenia and the observed association between this disorder and certain SNPs in genomic regions coding these transcripts further highlight the role of ncRNAs in the pathogenesis of schizophrenia (8). These transcripts have been shown to govern the complex pattern of gene expression, thus being considered as one of the principal epigenetic mechanisms of gene expression (8). It is worth mentioning epigenetic alterations in some brain areas and neural tracks characterize an important route through which environmental parameters interplay with personal genetic composition to influence susceptibility to psychiatric disorders during the lifespan (9). Moreover, two classes of ncRNAs namely microRNAs (miRNAs) and long non-coding RNAs (lncRNAs) are extremely abundant in the human brain, implying their crucial role in the appropriate function of this tissue (10, 11). While miRNAs mainly repress gene expression at post-transcriptional stage (12), lncRNAs can either enhance or suppress expression of gene through acting at chromatin, transcriptional an post-transcriptional levels (13). The important roles of these two classes of ncRNAs in the regulation of expression of genes and their developmental and tissue-specific signature suggest that they might underlie the observed aberrations in the transcriptome and proteome of brain tissue in patients with schizophrenia. Besides, alterations in the expression of lncRNAs might explain the plasticity of the organization of evolving neurons and their role in development of brain (14). In the current review, we explore the changes in the expression of miRNAs and lncRNAs in patients with schizophrenia. For the purpose of preparing this narrative review, we searched PubMed and google scholar with the key words “microRNA” or “miRNA” or “long non-coding RNA” or “lncRNA” AND “schizophrenia.” Subsequently, we assessed the relevance of the obtained material through reading the full texts of the articles. Finally, we tabulated the retrieved data in distinct tables.

## LncRNAs and Schizophrenia

LncRNAs are a huge and dissimilar group of ncRNA which have more than 200 nucleotides. LncRNAs signify the bulk of the non-coding transcriptome and utilize numerous mechanisms to exert their regulatory functions on gene expression among them being suppression or recruitment of transcription factors, modulation of chromatin structure and regulation of the stability of transcripts (15, 16). High throughput microarray investigations have demonstrated significant changes in lncRNA signature in the peripheral blood or post-mortem brain tissues of patients with schizophrenia compared with control samples (17–20).

## LncRNA Profile in Central Tissues

Expression of the lncRNA Gomafu has been regulated by neuronal activation. This lncRNA has been shown to directly interact with two splicing factors namely QKI and SRSF1. Therefore, aberrant expression of Gomafu changes the splicing patterns of DISC1 and ERBB4 genes to a pattern which is similar to what is reported in schizophrenia. Expression of Gomafu is substantially decreased in post-mortem cortical gray matter of patients with schizophrenia (20). Level of Gomafu activity has been correlated with neuronal structural plasticity which is strong in the course of development and subsequently is decreased in the adulthood (14). Therefore, abnormal activity of Gomafu in the brain tissues of subjects with schizophrenia might reflect abnormal neurodevelopment. Moreover, abnormal levels of this lncRNA might also affect human behavior, since it has been associated with susceptibility to substance abuse (21).

Hu et al. have assessed RNA profile of post-mortem brain samples in patients with schizophrenia and those with bipolar disorder and control subjects. They reported differential expression of 20 long intergenic non-coding RNAs (lincRNAs) in orbitofrontal cortex of bipolar patients and aberrant expression of 34 and 1 lincRNAs in anterior cingulate cortex and dorsolateral prefrontal cortex of patients with schizophrenia, respectively. Thus, they reported brain area-specific profiles for lincRNAs. Differentially expressed lincRNAs were enriched in pathways namely immune system development and oligodendrocyte differentiation. Moreover, they reported altered DNA methylation as a possible mechanism for dysregulation of lincRNAs (18).

## LncRNA Profile in Peripheral Blood

Chen et al. have examined expression profile of lncRNAs in the peripheral blood mononuclear cells (PBMCs) of patients with schizophrenia compared with healthy subjects. They reported differential expression of 125 lncRNAs between these two subgroups. Notably, expression levels of ENST00000394742, TCONS_l2_00025502, ENST00000563823, ENST00000521622, and TCONS_l2_00021339 were suggestively down-regulated in patients (17). LncRNA profiling has also revealed up-regulation of three lncRNAs in schizophrenia, down-regulation of six lncRNAs in major depressive disorder, and up-regulation of three lncRNAs in generalized anxiety disorder (GAD). Notably, lncRNAs observed to be up-regulated in schizophrenia were significantly decreased in patients with GAD. Furthermore, down-regulated lncRNAs in major depressive disorder were up-regulated in patients with schizophrenia. Finally, there were significant differences in the expression levels of lncRNAs between patients with schizophrenia and GAD. Therefore, a number of lncRNAs are putative biomarkers for differentiation of schizophrenia from major depressive disorder and generalized anxiety disorder (19). Sudhalkar et al. have examined expression levels of MEG3, PINT, and GAS5 in the PBMCs of patients with psychosis compared with healthy controls. They reported diagnostic differences with MEG3, PINT, and GAS5, and symptom acuity effect with MEG3 and GAS5. Moreover, there was significant difference in the expression of MEG3 between drug naïve patients and patients received risperidone (22). IFNG-AS1 expression has been shown to be down-regulated in patients with schizophrenia compared with healthy subjects in correlation with IFNG expression indicating a putative role for inflammation in this disorder (23). We have recently demonstrated down-regulation of FAS-AS1, PVT1, and TUG1 in patients with schizophrenia compared with controls. Yet, expressions of GAS5, NEAT1, and OIP5-AS1 were similar between patients and controls (24).

Table 1 exhibits the results of researches which demonstrated down-regulation of lncRNAs in schizophrenia.

**Table 1:** Down-regulated lncRNAs in schizophrenia (Empty cells show that this information has not been provided in the main articles).

**LncRNAs**	**Samples**	**Source**	**Targets/Regulators**	**Signaling pathways**	**Functional roles**	**References**
Gomafu	28 subjects with SZ and 28 non-psychiatric controls.	Fresh-frozen cortical gray matter from the superior temporal gyrus	DISC1, ERBB4	Alternative Splicing	Gomafu may be involved in regulating plasticity-related activity-dependent alternative splicing.	(20)
PINT	86 SCZ patients and 44 healthy controls were enrolled.	PBMC		Chronic inflammatory pathway	Expression of PINT was increased following exposure with LPS, but this effect was abolished with Risperidone.	(22)
GAS5					Expression of GAS5 was enhanced in response to LPS treatment.	
ENST00000394742	106 SCZ patients and 48 healthy controls were enrolled.	PBMC			These transcripts have been proposed as biomarkers for the diagnostic and prognostic applications.	(17)
TCONS_l2_00025502						
ENST00000563823						
ENST00000521622						
TCONS_l2_00021339						
IFNG-AS1	27 SCZ patients and 32 healthy controls were enrolled.	PBMC	IFNG	Inflammation related pathway	Inflammation and inflammatory LncRNAs might have a potential role in pathophysiology of Schizophrenia, and may be contributed to therapeutic approaches.	(23)
FAS-AS1	50 SCZ patients and 50 healthy controls were enrolled.	Blood			The association between FAS-AS1 expression and schizophrenia was remarkable in a subgroup of men.	(24)
PVT1					PVT1 and TUG1 were appropriate biomarkers in male patients.	
TUG1						

A high throughput microarray-based strategy has demonstrated 125 dysregulated lncRNAs in patients with schizophrenia compared with including 62 over-expressed and 63 under-expressed lncRNAs in patients. Antipsychotic treatment has resulted in reduction in NONHSAT089447 and NONHSAT041499 levels, parallel with decrease in the post-treatment Positive And Negative Syndrome Scale (PANSS) scores. Moreover, reduction in NONHSAT041499 levels have been associated with improvement of several clinical manifestations and better response to therapies (17).

Chen et al. have reported the effects of olanzapine treatment in suppression of expression of the NONHSAT089447 lncRNA. Small interfering RNA-mediated NONHSAT089447 silencing has reduced expression of dopamine receptors DRD3 and DRD5. In addition, Western blot studies verified the role of this lncRNA in regulation of DRD signaling (25). We have demonstrated up-regulation of HOXA-AS2, Linc-ROR, MEG3, SPRY4-IT1, and UCA1 in patients with schizophrenia compared with healthy subjects. Yet, when assessing their expressions in sex-based subclasses, the differences in their expressions were significant just among females. Moreover, we reported correlations between expressions of Linc-ROR and SPRY4-IT1 and age of patients (26). Ni et al. have profiled peripheral blood transcriptome of monozygotic twins discordant for schizophrenia. Using this approach, authors have demonstrated up-regulation of AC006129.1 lncRNA in patients. This lncRNA regulates inflammatory reactions through promoting expression of SOCS3 and CASP1. Further experiments showed that AC006129.1 interacts with the promoter region of the transcriptional repressor Capicua (CIC) to enhance the interactions of DNA methyltransferases with its promoter and decrease CIC expression, thus reversing CIC-associated SOCS3 and CASP1 suppression. Activation of SOCS3 increases the anti-inflammatory reactions by obstructing JAK/STAT pathway (27). Table 2 exhibits the list of up-regulated lncRNAs in schizophrenia.

**Table 2:** Up-regulated lncRNAs in schizophrenia (Empty cells show that this information has not been provided in the main articles).

**LncRNAs**	**Samples**	**Source**	**Targets/Regulators**	**Signaling pathways**	**Functional roles**	**References**
NONHSAT089447	40 SCZ patients and 40 healthy controls	PBMC	DRD3 and DRD5	Dopamine pathway	Dopamine receptors DRD3 and DRD5, and their downstream signals were activated by NONHSAT089447 expression.	(25)
NONHSAT041499	106 SCZ patients and 48 healthy controls	PBMC		Neuron apoptosis, learning, memory, behavior, sensory perception of sound, synapse organization and activity, layer formation in the cerebral cortex, stress-activated protein kinase signaling pathway and Ras protein signal transduction	ΔCT value of NONHSAT041499 was significantly higher in patients after the treatment, representing the substantial down-regulation of this lncRNA expression by the treatment. The symptomatology score and total score were meaningfully reduced following treatment.	(17)
NONHSAT098126					These transcripts have been suggested as markers for the diagnosis and prognostic evaluations.	
NONHSAT021545						
NONHSAT104778						
MEG3	86 SCZ patients and 44 healthy controls	PBMC		Chronic inflammatory pathway	Expression of MEG3 is lower in patients received risperidone treatment compared to those not receiving this drug.	(22)
THRIL	50 SCZ patients and 50 healthy controls	Blood			THRIL showed higher expression levels only in male subjects. This sex-based correlations imply the influence of sex hormones on its expressions.	(24)
HOXA-AS2 Linc-ROR MEG3 SPRY4-IT1 UCA1 MALAT1	60 SCZ patients and 60 healthy controls	Blood			HOXA-AS2, Linc-ROR, MEG3, UCA1, and SPRY4-IT1 are significantly up-regulated in total patients compared with total controls, but when evaluating in sex-specific manner, they only show significantly differences among female patients. There is also significant correlation between expression of HOXA-AS2, MALAT1, and UCA1 and age of participants in both patients and controls.	(26)
TCONS_00019174 ENST00000566208 NONHSAG045500 ENST00000517573 NONHSAT034045 NONHSAT142707	45 SCZ patients and 40 healthy controls	PBMC			The mentioned lncRNAs show lower levels in major depressive disorder in comparison with control group. But these lncRNAs show opposite trend in schizophrenia.	(28)
AC006129.1	157 SCZ patients and 134 healthy controls	Blood	SOCS3 and CASP1	Inflammatory response	AC006129.1 binds to the promoter of the transcriptional repressor Capicua, enhancing the interplay of DNA methyltransferases with the its promoter, thus amending CIC-induced SOCS3 and CASP1 suppression.	(27)

### Association Between LncRNA(s) SNP(s) and Risk of Schizophrenia

Few studies have appraised association between lncRNAs SNPs and risk of schizophrenia. Rao et al. have performed a two-phase association study on 8 tag SNPs that encompass the entire MIAT region in two independent cohorts from Han Chinese population. They demonstrated significant association between paranoid schizophrenia and the rs1894720. Moreover, there was a weak association between rs4274 and this condition. No specific haplotype was detected that modulate risk of paranoid schizophrenia in the assessed population (29).

### Diagnostic Value of LncRNAs in Schizophrenia

Expression levels of lncRNAs could be used as diagnostic markers for schizophrenia. We have assessed this possibility in a cohort of Iranian patients with schizophrenia (24, 26). Based on the obtained results in a limited number of patients, GAS5 and OIP5-AS1 have been proposed as appropriate biomarkers in female subjects (24). Table 3 summarizes the results of these studies.

**Table 3:** Diagnostic value of lncRNAs in schizophrenia.

**LncRNA**	**Samples**	**Validation in independent cohorts**	**Distinguish between**	**Area under curve (AUC)**	**Sensitivity**	**Specificity**	**References**
SPRY4-IT1	60 SCZ patients and 60 healthy controls	No	Female patients with schizophrenia from female controls	0.85			(26)
Combination of Linc-ROR, MEG3, SPRY4-IT1, and UCA1					95.2%	76.9%	
FAS-AS1	50 SCZ patients and 50 healthy controls	No	Diagnosis of schizophrenia in male subjects aged >50 years	0.825	90.48%	66.67%	(24)
GAS5			Diagnosis of schizophrenia in female persons	0.93	100%	86.96%	
			Diagnosis of schizophrenia in female persons aged <50		100%	100%	
NEAT1			Diagnosis of schizophrenia in female persons	0.86	86.67%	78.2%	
OIP5-AS1			Diagnosis of schizophrenia in female persons	0.87	100%	60.87%	
THRIL			Diagnosis of schizophrenia in female persons	0.817	86.67%	78.26%	
TUG1			Diagnosis of schizophrenia in male persons	0.832	71.43%	85.19%	
PVT1			Diagnosis of schizophrenia in male persons	0.83	76.47%	85.19%	

## miRNAs and Schizophrenia

These small transcripts have intricate temporospatial signature in the brain tissue and can modulate expressions of myriad of genes by working as the specificity elements for gene-silencing apparatus in the cells. Based on the observed association between miRNA dysregulation and substantial alterations in the network organization in the course of neurodevelopment, miRNAs are considered as important regulators of several neurological processes (30). Numerous studies have indicated aberrant expression of miRNAs in the brain and peripheral blood of patients with schizophrenia.

## miRNA Levels in Peripheral Blood

Lai et al. have used the same strategy to assess miRNA profile of PBMCs in patients with schizophrenia. They reported association between expression profile of 7 miRNAs and negative symptoms as well as neurocognitive performance scores (31). Lai et al. have demonstrated aberrant expression of a panel of miRNAs in patients with schizophrenia and correlation between expression pattern of some miRNAs and the presence of negative symptoms, level of neurocognitive function, and event-related potentials (31).

Gardiner et al. have assessed the miRNA signature in peripheral blood mononuclear cells (PBMCs) obtained from patients with schizophrenia and control individuals. Using microarray technique, they detected down-regulation of 33 miRNAs, dysregulation of seven of them being verified by real-time PCR technique as well. Notably, 17 down-regulated miRNAs have been shown to be transcribed from a particular imprinted locus at the maternally expressed DLK1-DIO3 area. This distinctive miRNA signature in PBMCs might represent a fundamental genetic or epigenetic mechanism for the pathogenesis of schizophrenia (32). Shi et al. have reported up-regulation of miR-181b, miR-219-2-3p, miR-1308, and let-7g whereas down-regulation of miR-195 in serum samples obtained from patients with schizophrenia compared with controls (33). Sun et al. have demonstrated over-expression of miR-132, miR-195, miR-30e, and miR-7 in plasma samples of patients with schizophrenia, and up-regulation of miR-212, miR-34a, and miR-30e in their PBMCs {#164}. They suggested that miRNA signature is more distinctive in plasma samples compared with PBMCs (34). A high throughput miRNA profiling revealed up-regulation of eight miRNAs in plasma samples obtained from schizophrenia patients, among them were miR-130b and miR-193a-3p which were up-regulated in schizophrenia but not in non-schizophrenia psychotic disorders. These results indicated these miRNAs as state-independent biomarkers for schizophrenia (35).

Lai et al. have subsequently assessed the impact of hospitalization on the expression levels of these miRNAs. Notably, expression of none of these miRNAs did not change after 2 months hospitalization of patients even when clinical symptoms were remarkably ameliorated. Thus, these miRNAs have been suggested as trait biomarkers instead of state-dependent biomarkers. Assessment of expression profile of hsa-miR-34a and hsa-miR-548d in post-mortem brain samples showed no difference between patients and controls (36).

## miRNA Profile in Central Tissues/Cell Lines

Expression profile of miRNAs has also been assessed in the olfactory epithelium as one of the limited available neural tissues that have neurons and neural stem cells. Mor et al. have detected over-expression of miR-382-5p in cultured olfactory cells obtained from schizophrenia patients compared with controls. Up-regulation of this miRNA was also verified in microdissected olfactory epithelium neuronal tissues of these patients. However, miR-382 was not expressed in lymphoblastoid cell lines originated from either patients with schizophrenia or control subjects. This miRNA has been shown to target FGFR1 and SPRY4, two genes whose expressions were decreased in the olfactory cells obtained from patients with schizophrenia (37).

**Figure 1:**
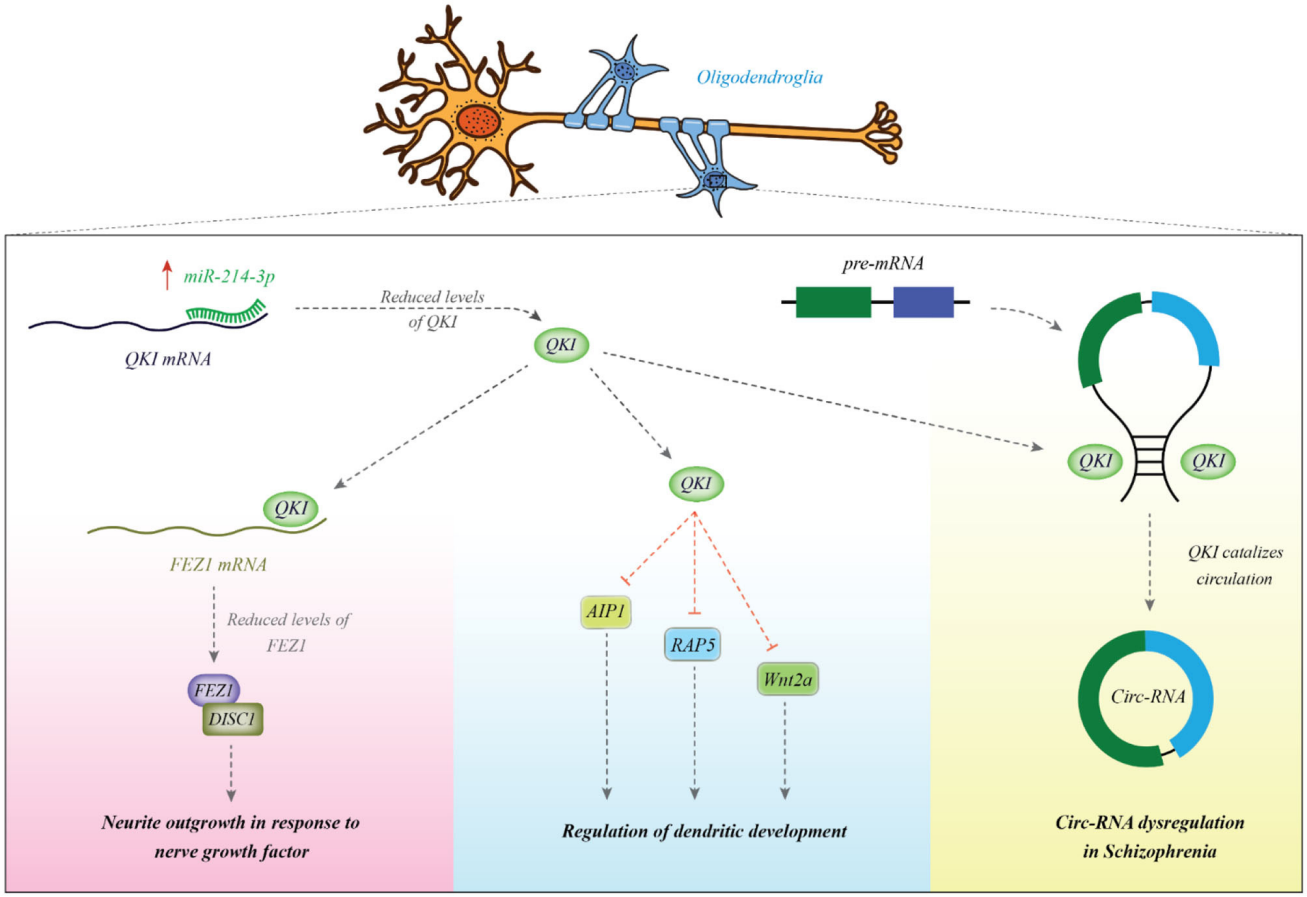
miR-214 is overexpressed in patients with schizophrenia. This miRNA binds with the 3′ UTR of Qki transcript to reduce its expression. Qki is an RNA-binding protein which modulates stability of several mRNAs among them are AIP1, Wnt2a, and Rab5. These transcripts are involved in the dendritic development (39). Qki can also bind with FEZ1 mRNA to enhance its stability (41). FEZ1 binds with DISC1 and increases neurite outgrowth in response to NGF (42). Qki can bind with intronic regions and participate in the biogenesis of circRNAs (43). Abnormal expression of Qki might be associated with the observed dysregulation of cricRNAs in schizophrenia (44).

Other experiments in HEK293 and SH-SY5Y cell lines have validated CALN1 as a target of miR-137 (38). A number of other miRNAs can also regulate development of dendrites, thus being implicated in the functionality of synapses and neuronal interactions. For instance, miR-214 has been shown to increase dendrite dimension, complexity and morphogenesis. Its involvement in the pathogenesis of schizophrenia has been suggested through the observed interaction between this miRNA and quaking (Qki), a candidate gene in schizophrenia. The miR-214-Qki axis is a fundamental axis in the modulation of dendritic development in the neurons (39). Experiments in a mouse neuroblastoma cell line (Neuro2A) have shown the regulatory role of miR-137 on the expression of several important proteins in the PI3K-Akt-mTOR axis which have functions downstream of neuregulin/ErbB and BDNF. Therefore, this miRNA controls neuronal reactions to these factors and dendritic development, thus contributing in the risk of schizophrenia (40). Figure 1 shows the mechanism of involvement of miR-214 in the pathogenesis of schizophrenia.

Tables 4, 5 show the list of down-/up-regulated miRNAs in the schizophrenia patients, respectively.

**Table 4:** Down-regulated miRNAs in schizophrenia [^&^Top targets based on the predictions of miRDB (http://www.mirdb.org/), empty cells show that this information has not been provided in the main articles].

**miRNAs**	**Samples**	**Cell line**	**Targets/Regulators**	**Signaling pathways**	**Function**	**References**
miR-137	–	Primary hippocampal and cortical neuron cultures made from C57BL/6J mouse embryos	PIK3R3, PTEN, RICTOR, and GSK3B, mTOR, p55γ, and Akt2	PI3K-Akt-mTOR pathway and Nrg/ErbB and BDNF signaling	miR-137 is required for Nrg/ErbB and BDNF signaling and participates in neurodevelopment.	(40)
miR-132	35 Schizophrenia (SCZ) patients and 34 healthy controls were enrolled.	Prefrontal cortical tissue	GATA2, PDE7B, ANKRD11, P250GAP, and FKBP2	PKA signaling pathway	miR-132 down-regulation in the dorsolateral prefrontal cortex is a feature of schizophrenia and 22q11 deletion, which causes schizophrenia-like symptoms and is associated with dysregulation of a number of miR-132 targets.	(45)
miR-26b miR-30b miR-29b miR-195 miR-92 miR-30a-5p miR-30d miR-20b miR-29c miR-29a miR-212 miR-7 miR-24 miR-30e miR-9-3p	13 Schizophrenia (SCZ) patients, 2 schizoaffective subjects, and 21 healthy controls were enrolled.	Prefrontal cortex	STRADB^&^ IRGQ^&^ PWWP2A^&^ CADM2^&^ SH3TC2^&^ DCUN1D3^&^ CDC73^&^ BRWD3^&^ COL5A3^&^ COL5A3^&^ CDK19^&^ RIMKLB^&^ CALD1^&^ CDC73^&^ SESN3^&^	Regulation of actin cytoskeleton, focal adhesion, MAPK signaling pathway, ECM-receptor interaction, phosphatidylinositol signaling, calcium signaling pathway, methionine metabolism, gap junction, tight junction, insulin signaling pathway, JAK-STAT signaling pathway, circadian rhythm.	DiGeorge critical region 8 (DGCR8), implicated in miRNA synthesis is positioned in a region (22q11) where microdeletions have been linked with higher risk of schizophrenia. DGCR8 variants that change expression or function of genes may participate in the etiology of schizophrenia by affecting miRNA synthesis and modulation of gene expression.	(46)
miR-432	90 Schizophrenia (SCZ) patients and 60 healthy controls were enrolled.	Mononuclear leukocytes	DAB2IP, PPP1R12B		Mononuclear leukocyte-based miRNA profiling is a possible way to recognize markers for schizophrenia.	(31)
miR-1306-3p	Neurons generated from induced pluripotent stem cell (iPSC) derived from 6 control subjects and 6 schizophrenia subjects.	Neurons produced from induced pluripotent stem cell (iPSC) derived from controls and SCZ.	GSK3B, CNTNAP1, DAO, GRIA1, GRIN1, GRIK3, and SLC17A7			(47)
miR-128						
miR-1306-5p						
miR-185-5p^*^						
miR-3175						
miR-3158-3p						
miR-185-3p^*^						
miR-486-3p^*^						
miR-1249						
miR-6840-5p						
miR-491-5p^*^						
miR-4804-5p						
miR-767-3p						

* *Shows miRNAs that have also been found to be differentially expressed in autopsy samples or peripheral cells in neuropsychiatric disorders*.

**Table 5:** Up-regulated microRNAs in schizophrenia (Empty cells show that this information has not been provided in the main articles).

**miRNA**	**Samples**	**Cell line**	**Targets/Regulators**	**Signaling pathways**	**Function**	**References**
miR-15 miR-26b miR-107 miR-181b	21 persons with schizophrenia and 21 non-psychiatric controls; DLPFC gray matter from 15 persons with schizophrenia and non-psychiatric controls	Fresh frozen post-mortem superior temporal gyrus (STG) gray matter tissues	DGCR8, BDNF, NRG1, RELN, DRD1, HTR4, GABR1, GRIN1, GRM7, CHRM1, and ATXN2.	Axon guidance, long-term potentiation, Wnt, ErbB, and MAPK signaling pathways	Schizophrenia is associated with a global increase in miRNA biogenesis and expression in the cerebral cortex and influences genes involved in the cortical structure and neural plasticity.	(48)
miR-382	20 Schizophrenia (SCZ) patients and 18 healthy controls were enrolled.	Olfactory Epithelium (OE)	FGFR1 and SPRY4	Fibroblast Growth Factor (FGF) signaling pathway	The higher levels of miR-382 expression in schizophrenia patients might be associated with the lower levels of FGFR1 and SPRY4 expression.	(37)
miR-214	Hippocampi were dissected from embryonic mice.	Hippocampal neurons	Qki	miR-214-Qki pathway	Protein levels of all Qki isoforms were reduced in miR-214-overexpressing cells. miR-214 also promotes dendritic but not axonal development in hippocampal neurons.	(39)
miR-181b	21 Schizophrenia (SCZ) patients and 21 healthy controls were enrolled.	Superior temporal gyrus	VSNL1 and GRIA2			(49)
miR-328 miR-17-5p miR-134 miR-652 miR-382 miR-107	74 Schizophrenia (SCZ) patients and 37 healthy controls were enrolled.	Dorsolateral prefrontal cortex	DICER, DROSHA and DGCR8	Melanogenesis, MAPK signaling pathway, T cell receptor signaling pathway, Axon guidance, Calcium signaling pathway, Long-term potentiation, Hypertrophic cardiomyopathy		(50)
miR-34a	35 Schizophrenia (SCZ) patients and 31 healthy controls were enrolled.	Dorsolateral prefrontal cortex	MAF1			(51)
miR-132^*^			NCR2			
miR-132			DCLRE1A			
miR-212			CXorf26			
miR-544			ATP2A2^&^			
miR-7			POLE4^&^			
miR-154^*^			ABCA4			
miR-106b	13 Schizophrenia (SCZ) patients, 2 schizoaffective subjects and 21 healthy controls were enrolled.	Prefrontal cortex	ENPP5^&^			(46)
miR-137	–	Barrel cortex	Dusp1, Egr2, Dusp4, Ptgs2, and Sgk1	Glucocorticoid receptor–dependent signaling network	Decreased brain miR-137 levels may lower the risk of schizophrenia-related behavior.	(52)
	–	SH-SY5Y cell lines	CALN1		Expression of CALN1 is inhibited by miR-137. CALN1 may be down-regulated in schizophrenia patients.	(38)
miR-34a miR-449a miR-564 miR-548d miR-572 miR-652	In total, 90 Schizophrenia patients and 60 healthy subjects were enrolled.	Mononuclear leukocytes	DDX17, DLL1, INF2, JAG1, DAB2IP		Mononuclear leukocyte-based miRNA signature is an achievable method to find biomarkers for schizophrenia.	(31)
			DDX17, DLL1, INF2, JAG1, PPP1R12B			
			SIK3^&^			
			CREBBP^&^			
			TOAK2^&^			
			CSAG1^&^			
miR-34b-3p^*^	6 control subjects and 6 schizophrenia subjects.	Neurons generated from induced pluripotent stem cell (iPSC) derived from controls and SCZ.	DISC1, GSK3β, MYT1L, TCF7L2, CNTNAP1, NRXN1, GRM3, GRIN2A, GRIN2B, GRIN2D, GRIK2, GRIK3, CCK, GABRA1 GRIN2B, GABBR2, and GABRB2	glutamatergic transmission and GABAergic transmission		(47)
miR-34c-5p^*^						
miR-26b-5p^*^						
miR-146b-3p^*^						
miR-23a-5p^*^						
miR-296-3p^*^						
miR-4449^*^						
miR-4792						
miR-148a-3p						
miR-320b						
miR-3609						
miR-320c						
miR-126-3p^*^						
miR-320e						
miR-7704						
miR-181b-5p^*^						
miR-146a-5p^*^						
miR-6757-5p						
miR-4682						
miR-26a-5p^*^						
miR-3195						
miR-126-5p^*^						
miR-125a-5p						
miR-548q						
miR-320d						
miR-4497						
miR-27a-3p^*^						
miR-455-5p						
miR-7113-5p						
miR-6842-5p						
miR-146b-5p						
miR-6852-5p						
miR-7	50 Schizophrenia (SCZ) patients and 50 healthy controls were enrolled.	Plasma	SHANK3	miR-7/Shank3	miR-7 binds with the 3′ UTR of SHANK3 mRNA and causes the alteration of neuronal morphology and function.	(53)
miR-30e	61 Schizophrenia (SCZ) patients and 62 healthy controls were enrolled.	Plasma	CDC73^&^		Profile of these miRNAs is useful non-invasive method for diagnosis of schizophrenia, assessment of symptom improvements, therapeutic responses and evaluation of prognosis.	(54)
miR-181b			PLCXD3^&^			
miR-34a			MSR1^&^			
miR-346			PGK1^&^			
miR-7			RIMKLB^&^			
miR-132	25 Schizophrenia (SCZ) patients and 13 healthy controls were enrolled.	Plasma	CDK19^&^			(34)
miR-195			CADM2^&^			
miR-30e			CDC73^&^			
miR-7			RIMKLB^&^			
miR-212		PBMC	CDK19^&^			
miR-34a			MSR1^&^			
miR-30e			CDC73^&^			
miR-1273	82 Schizophrenia (SCZ) patients and 43 healthy controls were enrolled.	PBMC	FGF9^&^		After treatment with antipsychotic drugs, miR-21 expression level but no other miRNAs had significantly decreased.	(55)
miR-1303			OGFRL1^&^			
miR-21			STK38L^&^			
miR-3064-5p			ZFAND3^&^			
miR-3131			TRPS1^&^			
miR-3687			–			
miR-3916			NAV3^&^			
miR-4428			EPHB1^&^			
miR-4725-3p			PIK3R3^&^			
miR-5096			–			
miR-206	In total, 149 Schizophrenia (SCZ) patients and 146 healthy controls were enrolled.	Blood exosomes	BDNF, GALNT15, CDC42, and DISC1	Protein glycosylation, neurodevelopment, neurotransmission, and synaptic plasticity	Blood exosomal miRNAs are promising biomarkers for SCZ.	(56)
miR-145-5p						
miR-133a-3p						

& *Top targets based on the predictions of miRDB (http://www.mirdb.org/)*.

* *Shows miRNAs that have also been found to be differentially expressed in autopsy samples or peripheral cells in neuropsychiatric disorders*.

## miRNAs Variations in Schizophrenia

Some studies have verified associations between SNPs within miRNA-coding genes and risk of schizophrenia. For instance, in a genome-wide association study, Ripke et al. have shown significant association between the rs1625579 of the miR-137 and risk of schizophrenia (57). Notably, others have confirmed associations between miR-137 target genes *CUB, CSMD1, C10orf26, CACNA1C, TCF4*, and *ZNF804A*, and risk of schizophrenia (58–61). Notably, the mentioned SNP in miR-137 has been shown to influence cognitive activity. The T/T genotype of this SNP is correlated with working memory defects in patients with schizophrenia as reflected by their reduced scores in the brief assessment of cognition in schizophrenia instrument (62). The risk genotype has also been associated with dorsolateral prefrontal cortex hyperactivation (DLPFC) in patients with schizophrenia (63). In addition, the risk allele of this SNP has been associated with down-regulation of miR-137 in schizophrenia and is potentially involved in the modulation of expression of the schizophrenia risk locus TCF4, more emphasizing on the participation of miR-137 and its downstream molecules in this disorder (64). These studies further support the role of this miRNA in the pathogenesis of schizophrenia.

## Diagnostic Value of miRNAs in Schizophrenia

Diagnostic value of miRNAs has been assessed in different biological sources of patients with schizophrenia using statistical methods, such as plotting the receiver operating characteristic curve and calculation of the area under the curve (AUC) values. Such method has shown the diagnostic values of 0.767 and 0.756 for plasma and PBMC expression patterns of miR-30e, respectively. Plasma levels of this miRNA had sensitivity and specificity of 90.90 and 60.00%, respectively. These values for its PBMC levels were 81.80 and 68.00%, respectively. Moreover, logistic regression analysis showed higher sensitivity of plasma levels of this miRNA in differentiating schizophrenia patients from healthy subjects compared with its PBMC levels (34). The diagnostic value of a panel of over-expressed miRNAs including miR-30e, miR-181b, miR-34a, miR-346, and miR-7 has been reported to be 0.713. Notably, suitable pharmacotherapy resulted in down-regulation of miR-132, miR-181b, miR-432, and miR-30e expressions. Besides, there was a remarkable correlation between amelioration of clinical symptoms and alterations in the expression levels of miR-132, miR-181b, miR-212, and miR-30e (54). A certain molecular axis including the transcription factor the early growth response protein 1 (EGR1), miR-30a-5p, and its target gene NEUROD1 has been shown to differentiate schizophrenia patients from healthy subjects with diagnostic accuracy of 0.962 which was far higher than the diagnostic power of miR-30a-5p alone. This axis has also been proved useful for monitoring of patients in acute psychotic phase (65). Table 6 shows the results of studies which assessed diagnostic role of miRNAs in the schizophrenia.

**Table 6:** Diagnostic role of miRNAs in schizophrenia.

**miRNA**	**Samples**	**Validation in independent cohorts**	**Distinguish between**	**Area under curve (AUC)**	**Sensitivity (%)**	**Specificity (%)**	**References**
miR-30e (Plasma) miR-30e (PBMC)	25 schizophrenia patients and 13 healthy controls were enrolled.	No	Differentiate schizophrenia patients from normal controls	0.767 0.756	90.90% 81.80%	60.00% 68.00%	(34)
miR-30e, miR-181b, miR-34a, miR-346, and miR-7	61 schizophrenia patients and 62 healthy controls were enrolled.	No		0.713	35.5%	90.2%	(54)

## Discussion

Schizophrenia is complex disorder caused by interaction between several genomic loci. The speculation that this disorder is caused by one or a few common principal gene effects has been experimentally examined in genome-wide linkage studies yet results generally showed no genome-wide significance (66). In addition to genomic variants that contribute in the pathogenesis of this disorder, some other mechanisms might modify the risk of development of schizophrenia. Expressions of ncRNAs are modulated by neuronal activation (20), suggesting a role for these transcripts in the pathophysiology of neuropsychiatric disorders. In the current study, we reviewed the literature about the role of ncRNAs in the pathophysiology of schizophrenia. Based on the above-mentioned evidence, several lncRNAs and miRNAs have been dysregulated in blood or brain samples of patients with schizophrenia. Not discounting the role of ncRNAs as biomarkers for schizophrenia, peripheral expression profile of these transcripts does not necessarily reflect their expression in the brain tissues (36). Aberrant expression of a number of these transcripts has been associated with PANSS score (17). Moreover, clinical diagnoses of psychosis and symptom severity have been shown to alter expression of a number of lncRNAs (22) indicating a substantial role for these transcripts in the pathogenesis of schizophrenia.

Although there is a considerable level of overlap between psychiatric disorders in the terms of contributing genetic factors, expression levels of some ncRNAs could be used to differentiate a number of these conditions (19). Moreover, expression profile of certain ncRNAs can distinguish patients with schizophrenia from healthy subjects. At the present, schizophrenia is principally diagnosed based on the clinical symptoms and signs instead of on the pathophysiological biomarkers (67). Identification of such biomarkers would facilitate detection of malingering, thus has practical significance in the forensic medicine in the assessment of cases pretending psychological disorders for a particular gain. Moreover, molecular biomarkers have implications in the establishment of targeted therapies. Notably, expression profile of ncRNAs in the circulation has the potential to improve the diagnostic and prognostic assessment of patients with schizophrenia (67). A former study has demonstrated up-regulation of NONHSAT089447, NONHSAT021545, and NONHSAT041499 lncRNAs in patients with schizophrenia, while down-regulation of these transcripts in patients with GAD. Notably, authors have reported six lncRNAs with opposite expression patterns in schizophrenia and major depressive disorder. Moreover, they have identified three GAD-related lncRNAs whose expressions were significantly differences between patients with schizophrenia and GAD patients (28). However, most of these studies do not test if expression profile of ncRNAs can distinguish people with schizophrenia from people with other disorders, such as major depression, bipolar disorder, or autism. Based on the similarities in many of clinical symptoms between subjects with schizophrenia and other disorders, including bipolar disorder, autism, and major depression, identification of specific markers for each disease has practical significance. Therefore, this field should be explored in future investigations.

Particularly, miRNA signature has been correlated with clinical course, patients' response to pharmacologic interventions and prognosis of patients with schizophrenia (54). Perhaps, the most intensively assessed miRNA in this regard is miR-30 family. Consistent with this observation, expression of EGR1 which regulates expressions of this miRNA family has been decreased in PBMCs of patients with schizophrenia. On the other hand, expression of NEUROD1 as a target gene of miR-30a-5p has been increased in these patients (65). Based on the results of this study, identification of transcription factor/ miRNA/ target gene axes would be a practical method for recognition of molecular pathways in the pathogenesis of schizophrenia and development of diagnostic/ prognostic panels for this disorder.

Several schizophrenia-associated ncRNAs have been shown to modulate immune responses and inflammatory pathways. Recent studies have highlighted the presence of intricate interplay between the immune system, systemic inflammatory responses, and the central nervous system, which can result alterations in mood, cognitive functions, and behavior. All of these aspects contribute in the pathogenesis of schizophrenia (68). Immune responses can influence activity of neurotransmitters as well as neurodegenerative and neurodevelopmental processes all of which are related with this disorder (68). Assessment of lncRNA signature in Amygdala samples from schizophrenia patients has further endorsed dysregulation of immune-associated lncRNAs in these samples (69). Thus, the functional axes between ncRNAs and immune-related genes provide an explanation for involvement of these transcripts in the pathophysiology of schizophrenia.

Besides, high throughput studies in schizophrenia subjects have shown a tendency toward global up-regulation of miRNAs normally abundant in infants, whereas down-regulation of those normally abundant in prepuberty. Therefore, dysregulation of miRNAs dynamic changes might be involved in the pathogenesis of schizophrenia (70). A number of ncRNAs might explain the observed dissimilarity in the brain activation modes between patients with schizophrenia and controls. For instance, Gomafu has been shown to be intensely regulated in reaction to neuronal activation (71). This lncRNA is also implicated in schizophrenia-related alternative splicing (71). Therefore, aberrant expression of this lncRNA might reflect the abnormal brain activity in these patients.

Finally, it is worth mentioning that miRNAs have distinctive roles as trait-dependent markers or state-dependent markers. This speculation is supported by amelioration of dysregulated expression of a number of miRNAs after pharmacotherapy (54), while no change in the expression of other miRNAs following suitable treatments (36). Therefore, it is necessary to define the role of each miRNA as trait- or state-dependent marker to design distinctive diagnostic/ prognostic miRNA panels for schizophrenia.

Taken together, lncRNAs and miRNAs are potential transcripts that can explain the difference in expression profile of protein coding genes in brain and blood tissues of patients with schizophrenia and healthy subjects. Moreover, as a number of above-mentioned ncRNAs are functionally related with dopamine neurotransmission, these ncRNAs might alter response of patients to some types of antipsychotic drugs. In addition, several ncRNAs can be used as disease markers in schizophrenia.

Several questions should be addressed about the role of ncRNAs in the development of schizophrenia. Studies reviewed in this article have mostly assessed expression of ncRNAs in adult patients. Although differentially expressed ncRNAs among patients and controls have been enriched in neurodevelopment, neurotransmission, and synaptic plasticity, the functional impact of these ncRNAs in the development of neurons should be assessed through knock-out/-in studies in animal models and cell lines.

Finally, most of studies reviewed here have not appraised the effects of antipsychotic drugs on expression of ncRNAs. The data regarding the therapeutic regimens and types of antipsychotic drugs have not been presented in the main articles. This is possibly because patients have been under treatment with different antipsychotic drugs. We mention this point as a limitation of these studies.

## Data Availability Statement

The original contributions presented in the study are included in the article/supplementary material, further inquiries can be directed to the corresponding author/s.

## Author Contributions

All authors listed have made a substantial, direct and intellectual contribution to the work, and approved it for publication.

## Conflict of Interest

The authors declare that the research was conducted in the absence of any commercial or financial relationships that could be construed as a potential conflict of interest.
